# Genome‐wide association study of periodontitis severity and progression

**DOI:** 10.1002/jper.70017

**Published:** 2025-12-17

**Authors:** Flavia Teles, Ganesh Chandrasekaran, Lynn Martin, Poojan Shrestha, Kevin Moss, Michele Patel, Michael J. Kallan, Camila Furquim, Andrew J. Cucchiara, James D. Beck, Kari E. North, Joseph Glessner, Kimon Divaris

**Affiliations:** ^1^ Department of Basic & Translational Sciences School of Dental Medicine University of Pennsylvania Philadelphia Pennsylvania USA; ^2^ Center for Innovation and Precision Dentistry University of Pennsylvania Philadelphia Pennsylvania USA; ^3^ Department of Biostatistics Epidemiology and Informatics, Perelman School of Medicine, University of Pennsylvania Philadelphia Pennsylvania USA; ^4^ Department of Pediatric Dentistry and Dental Public Health Adams School of Dentistry University of North Carolina‐Chapel Hill Chapel Hill North Carolina USA; ^5^ Department of Biostatistics and Health Data Sciences School of Medicine Indiana University Indianapolis Indiana USA; ^6^ Department of Biomedical Sciences Adams School of Dentistry University of North Carolina‐Chapel Hill Chapel Hill North Carolina USA; ^7^ The Forsyth Institute Cambridge Massachusetts USA; ^8^ Center for Clinical Epidemiology and Biostatistics Perelman School of Medicine University of Pennsylvania Philadelphia Pennsylvania USA; ^9^ Department of Periodontology and Oral Implantology Dental Research Division University of Guarulhos Guarulhos São Paulo Brazil; ^10^ Department of Periodontology, Endodontics, and Dental Hygiene Adams School of Dentistry University of North Carolina‐Chapel Hill Chapel Hill North Carolina USA; ^11^ Border Health Research Center School of Public Health UTHealth Houston Brownsville Texas USA; ^12^ Department of Internal Medicine McGovern Medical School UTHealth Houston Houston Texas USA; ^13^ Department of Epidemiology Gillings School of Global Public Health University of North Carolina‐Chapel Hill Chapel Hill North Carolina USA; ^14^ Children's Hospital of Philadelphia Philadelphia Pennsylvania USA

**Keywords:** disease progression, genes, genome‐wide association study, periodontitis, phenotype, polymorphism, single nucleotide, susceptibility

## Abstract

**Background:**

To add to the knowledge base of periodontal genomics, we carried out a genome‐wide association study (GWAS) of periodontitis severity and progression among 416 mixed‐ethnicity adult participants of a periodontitis clinical study.

**Methods:**

Participants were 168 adults (mean age = 50 years, 46% males) with severe periodontitis and 248 adults (mean age = 48 years, 40% males) without severe periodontitis, including 147 with mild periodontitis and 101 periodontally healthy. Disease progression information over a 12‐month period was available for 368 of these participants. Single marker discovery analysis relied on logistic regression models adjusted for age, sex, and genetically determined ancestry using a conventional *p *< 5x10^−8^ genome‐wide statistical significance criterion. Genome‐wide significant loci were annotated and examined for associations with periodontal disease traits in external cohorts of 10,019 Hispanic/Latinos, 4,554 European Americans, and 973 African Americans.

**Results:**

All GWAS single nucleotide polymorphisms (SNPs) explained 34% of phenotypic variance between periodontitis cases and controls and 57% of the variance in disease progression in this study. We identified 2 genome‐wide significant loci associated with disease progression (*SUMO2P2*, small ubiquitin‐like modifier 2, rs72691774, *p* = 1.9x10^−8^] and *CUBN* (cubilin, rs565051161, *p* = 3.9x10^−8^). *CUBN* was strongly associated with periodontal disease in the independent samples of African Americans (rs7082270, *p *= 3.1x10^−7^) and Hispanic/Latinos (rs1276710, *p* = 1.5x10^−5^), albeit the lead SNPs were rare and differed in each population. Meanwhile, *ZBTB16* (zinc finger and BTB domain‐containing 16) showed the strongest evidence of association with severe periodontitis (rs454802, *p *= 2.2x10^−7^).

**Conclusions:**

This study's results emanate from a well‐characterized cohort of periodontitis severity and progression and add to the knowledge base of periodontal genomics and the underlying individual disease susceptibility.

**Plain language summary:**

This study assessed the association of gene variants in association with gum disease severity and progression in 416 participants of a clinical study. Participants were 168 adults (mean age = 50 years, 46% males) with severe disease and 248 adults (mean age = 48 years, 40% males) without severe disease, including 147 with mild disease and 101 without disease. Disease progression information over a 12‐month period was available for 368 of these participants. Single marker discovery analysis relied on logistic regression models adjusted for age, sex, and genetically determined ancestry. Genome‐wide significant loci were annotated and examined for associations with periodontal disease traits in external cohorts of 10,019 Hispanic/Latinos, 4,554 European Americans, and 973 African Americans. All gene variants explained 34% of the variance between cases and controls and 57% of the variance in disease progression in this study. We identified 2 genome‐wide significant loci associated with disease progression (*SUMO2P2* and *CUBN*). *CUBN* was strongly associated with periodontal disease in the independent samples of African Americans and Hispanic/Latinos. *ZBTB16* showed the strongest evidence of association with severe periodontitis. This study's results emanate from a well‐characterized cohort of periodontitis severity and progression, suggest that about one‐third of variance in disease severity and over half of variance in disease progression are attributable to individual susceptibility, and add to the knowledge base of periodontal genomics.

## 1 INTRODUCTION

Periodontitis is a multifactorial disease, caused by a complex interaction between the oral microbiome and aberrant inflammatory host response.^1^ The existence of a genetic underpinning of periodontitis is supported by several lines of investigation, including twin and family studies, candidate gene studies, and most recently, agnostic scans of the human genome, that is, genome‐wide association studies (GWAS).^2^ The advent and democratization of next‐generation sequencing has facilitated the conduct of several independent GWAS for dental, oral, and craniofacial diseases. In principle, evidence of association generated by these studies is unbiased or at least not restricted by a priori hypotheses. Despite this new body of evidence, there are few consensuses and replicable genetic risk loci for periodontitis.^3^ Nevertheless, there is great potential for high‐quality genetic association information to accelerate discovery and translation. New candidates nominated by GWAS, when validated in independent populations and followed by functional characterization and experimental validation, have the potential to reveal new biological mechanisms underlying periodontitis pathogenesis, as is the recent case with *PLG*.^4^ Moreover, consensus, validated genetic markers (e.g., single nucleotide polymorphisms [SNPs]) for periodontitis can form the basis of polygenic risk scores (PRS) that will likely be suitable for clinical applications in the future.^5^ ^,^ ^6^

The current genomics evidence base for periodontitis is limited by variation and imprecision in clinical definitions of periodontitis employed in GWAS, relatively small sample sizes in most studies, and lack of ancestrally diverse studied populations.^7^ While sample size and population characteristics may not be amenable in most studies, there may be opportunities to improve upon the selection of disease features and phenotype definitions to be interrogated. This is important because studies of early onset^8^ or aggressive disease^9^ are more likely to identify risk loci that confer genetic liability for periodontitis. Moreover, these loci (e.g., *SIGLEC5*, initially identified for aggressive periodontitis) may be relevant for the more common forms of periodontitis found in large population‐based samples.^10^ It can be argued that focusing GWAS periodontitis phenotypes on relatively severe traits according to age, clinical presentation, or both^11^ is a promising strategy for identifying disease‐relevant genetic signals. Here, we leveraged an unprecedented opportunity to carry out a GWAS in a small but well‐characterized sample of adults with severe periodontitis cases and controls, that was clinically followed for a 12‐month period to assess periodontitis progression. Accordingly, the objective of this study was to add to the knowledge base of periodontal genomics by identifying genomic loci associated with severe periodontitis and periodontitis progression.

## 2 MATERIALS AND METHODS

### 2.1 Study design and population

This GWAS was carried out in the context of prospective longitudinal multicenter study of periodontitis “Biomarkers of Periodontal Disease Progression.” The four centers of the parent study included The Forsyth Institute (Cambridge, MA), New York University College of Dentistry (New York, NY), Southern Illinois University School of Dental Medicine (Alton, IL), and the State University of New York at Buffalo (Buffalo, NY). The study was undertaken in accordance with the Helsinki Declaration and was approved by the Institutional Review Board at each participating center. All study participants provided written informed consent. A detailed description of the study, as well as inclusion and exclusion criteria can be found in the study's clinical trial registration at clinicaltrial.gov (NCT01489839). In brief, after enrollment, participants were monitored clinically for up to 1 year every 2 months to identify periodontal sites and individual participants experiencing periodontal disease progression. Details regarding recruitment, monitoring, and patterns of periodontitis progression have been published elsewhere.^12^

### 2.2 Clinical data

Participants had periodontal parameters measured at up to 168 sites: at 6 sites per tooth—mesiobuccal, buccal, distobuccal, mesiolingual, lingual, and distolingual—for up to 28 teeth (excluding third molars) probing depth (PD); measurement of distance from the cementoenamel junction (CEJ) to the free gingival margin (B measure) (in case of recession, a negative value was assigned); clinical attachment loss (CAL, calculated by subtracting the B measure from the PD); presence or absence of plaque, gingival redness, bleeding on probing (BOP), and suppuration were determined. PD and the B measure were measured using calibrated North Carolina manual periodontal probes* rounding down to the nearest millimeter. PD and the B measure were measured twice at pre‐molars and the first and second molars. CAL was calculated for each pass by the electronic data capturing (EDC) system. If the difference between the 2 measurements was ≥2 mm, the EDC prompted the examiner to obtain PD and the B measure a third time. The median CAL among the 2 or 3 passes was used for analysis. All participants who completed the 12‐month monitoring phase attended at least 6 of 7 monitoring visits. To be included in analyses involving periodontitis severity, participants had to have completed at least the baseline study visit.

### 2.3 Phenotype definitions

#### 2.3.1 Periodontitis severity

According to the study's inclusion criteria, participants with severe periodontitis had ≥8 teeth with ≥1 site with PD ≥5 mm and concomitant CAL ≥3 mm. Those with mild periodontal disease had ≥4 teeth with ≥1 site with PD ≥5 mm and concomitant CAL ≥2 mm. To be included in the periodontitis group, participants also had to present radiographic evidence of alveolar bone loss around at least 2 of the affected teeth. Periodontally healthy subjects had no radiographic evidence of alveolar bone loss and all teeth with PD of ≤3 mm irrespective of CAL; PD ≥4 mm with no CAL (except for the distal of the second molars); or, for distal of second molars, PD = 4 mm with concomitant CAL ≤2 mm. For the primary GWAS of periodontitis severity, we contrasted participants with severe periodontitis against those without severe periodontitis (i.e., mild disease and periodontally healthy). For secondary supplemental analyses, participants were re‐classified in 3 categories according to the 2018 World Workshop^13^ criteria: periodontally healthy (*n* = 113), stage II periodontitis (*n* = 34), and stage III (*n *= 268) periodontitis, with the latter 2 groups comprising the periodontitis cases.

#### 2.3.2 Disease progression

Disease progression was defined based on longitudinal changes in CAL as previously reported.^12^, ^14^ First, linear mixed models were fitted to longitudinal CAL measurements for each measured site, and modelled CAL values were then used to categorize sites according to their progression.^14^ Subsequently, study participants were stratified into the following 4 progression groups: P_0 _= participants with no sites progressing; P_1 _= participants with 1–2 sites progressing; P_2 _= participants with ≥3 sites progressing. According to the study protocol, participants with ≥6 sites with cumulative CAL ≥2 mm compared to the baseline had their monitoring phase interrupted and proceeded to treatment. In the present study they were categorized as P_3_, that is, most progressing. To be included in analyses involving disease progression, participants had to have completed the 12‐month monitoring phase, attended at least 6 of 7 monitoring visits, and be examined by the same examiner in all visits.

### 2.4 Serum sample collection and genotyping

Study participants abstained from brushing teeth, chewing gum, eating, or drinking for at least 1½ h before study visits. At baseline, 10 mL of blood was collected in a serum tube with a clot activator†. After centrifuging, serum was aliquoted (250 µL/tube) and aliquots were snap frozen and transferred to a −80°C freezer until analysis. Genotyping was carried out using an array offering ∼660K genetic markers‡. To minimize the impact of batch effects and associated biases, samples were randomly plated without consideration of their periodontal status or disease progression, which were unknown at the time of processing.

### 2.5 GWAS

#### 2.5.1 Discovery analysis

Genotype imputation was performed using established pipelines and quality control procedures^15^ available via the University of Michigan Imputation Server, using the 1000 Genomes panel (Phase3 v5) comprising 5,008 haplotypes from 26 world‐wide populations as a reference. After exclusion of monomorphic SNPs, those with Hardy–Weinberg equilibrium *p* < 5x10^−6^ and minor allele frequency (MAF) < 1%, 9,604,584 were retained and carried forward to analyses. Heritability (i.e., phenotypic variance explained by all GWAS SNPs, *h^2^*) was estimated using Genome‐wide Complex Trait Analysis (GCTA)^16^ while adjusting for age, sex, and 3 ancestry principal components (PCs). Single marker (i.e., SNP) discovery analysis relied on logistic and linear regression models (i.e., for severity and progression, respectively) criterion implemented in PLINK,^17^ adjusting for age, sex, and 3 ancestry PCs and using a conventional *p* < 5.0x10^−8^ genome‐wide statistical significance threshold. Gene‐based analyses were carried out using MAGMA^18^ and a Bonferroni corrected *p*‐value criterion of 0.05/19659 = 2.5x10^−6^. Loci showing the strongest evidence of association (i.e., genome‐wide significant ones, as well as all with SNPs and genes with suggestive evidence of association marked by *p* < 10^−6^ and *p* < 10^−4^, respectively) with periodontitis diagnosis and progression were annotated in terms of genomic context, known associations, predicted function using Combined Annotation Dependent Depletion (CADD)^19^ and Regulome DB^20^ scores, and were visualized using Locus Zoom.^21^

#### 2.5.2 Generalization to external cohorts and examination of previously reported associations

To determine whether any identified genome‐wide significant loci showed evidence of association with periodontal traits in external, independent cohorts with dental and genetic information, we used publicly available data comprising 10,019 Hispanic/Latinos from the HCHS/SOL study,^22^ and 4,554 European Americans and 973 African Americans from the Dental ARIC study.^23^ The 2 traits interrogated in the external cohorts were a binary classification of whether a participant was within the top quintile (i.e., top 20%) of mean person‐level attachment loss (CAL) among similarly‐aged individuals, that is, within 5‐year age groups^11^ and the number of remaining natural teeth. We use the term generalization instead of replication because the traits examined in the discovery and the external cohorts (i.e., periodontitis progression, increased attachment loss, and tooth loss) while related, are distinct. We considered that the association of a genome‐wide significant locus generalized if individual SNPs associations in our study and the external cohorts were directionally consistent and met Bonferroni‐corrected statistical significance criteria or if other adjacent SNPs within (i.e., intronic to) the same locus met Bonferroni‐corrected statistical significance criteria. Finally, to maximize insights gained from this study's results, we examined markers previously reported as genome‐wide significantly associated with periodontitis in the NHGRI‐EBI GWAS catalogue^24^ and recent reports,^25^ as well as those in loci with previously reported mechanistic evidence of association, that is, *IL37* ^26^ and *PLG*,^4^ for any evidence of association in our data. In the examination of these associations, we considered a Bonferroni multiple testing correction. that is, the critical p‐value threshold accounting for 30 SNPs tested for association with 3 traits were 0.05/90 = 5.6x10^−4^.

## 3 RESULTS

Demographic characteristics and periodontitis classification of the 416 individuals included in this study are presented in Supplemental Table S1 (Table S1 in the online Journal of Periodontology). Most were non‐Hispanic whites, and their mean age was 48.4 years. About one‐quarter of participants (*n* = 101) were classified as periodontally healthy, with the remaining having mild periodontitis (*n* = 147, 35.3%) or severe periodontitis (*n* = 168, 40.4%). When classified according to 2018 WW criteria, most periodontitis cases (*n* = 280, 67.3% of total) were stage III. In terms of progression, 44.7% of participants had no progressing sites, and smaller proportions had 1–2 sites (23.3%) or ≥3 sites (11.5%) progressing. Thirty‐seven participants (8.9%) were in the highest progression group, that is, had their monitoring phase interrupted and entered rescue therapy. Table 1 and Table 2 present participants’ demographic characteristics and clinical data across strata of periodontal diagnoses at baseline and periodontitis progression group. As expected, periodontally‐healthy participants were younger and more likely to be female compared to those in the periodontitis groups and had more favorable measures of dental and periodontal health (e.g., fewer missing teeth, less mean PD, CAL, plaque, and BOP scores). Similar patterns were evident among those included in the disease progression analysis, with more progression associated with worse baseline oral health measures.

**TABLE 1:** Demographic and clinical characteristics of periodontitis diagnosis groups at the baseline visit.

	Study‐defined enrollment criteria			2018 World Workshop criteria^13^		
Parameter	No disease	Mild disease	Severe disease	No disease	Stage II	Stage III
Entire sample, *n* = 416 (%)	101 (24.3)	147 (35.3)	168 (40.4)	101 (24.3)	35 (8.4)	280 (67.3)

	*n* (%)	*n* (%)	*n* (%)	*n* (%)	*n* (%)	*n* (%)
**Sex**						
Male	30 (29.7)	69 (46.9)	77 (45.8)	30 (29.7)	7 (20.0)	139 (49.6)
**Age**						
Years, mean (SD)	39.0 (12.3)	53.2 (11.9)	49.9 (12.0)	39.0 (12.3)	52.3 (13.1)	51.3 (11.9)
**Race**						
African American	17 (16.8)	25 (17.0)	60 (35.7)	17 (16.8)	4 (11.4)	81 (28.9)
White	67 (66.3)	96 (65.3)	99 (58.9)	67 (66.3)	26 (74.3)	169 (60.4)
Asian	15 (14.9)	9 (6.1)	3 (1.8)	15 (14.9)	2 (5.7)	10 (3.6)
American Indian / Alaskan Native	0 (0.0)	2 (1.4)	0 (0.0)	0 (0.0)	0 (0.0)	2 (0.7)
More than 1 race	1 (1.0)	5 (3.4)	2 (1.2)	1 (1.0)	2 (5.7)	5 (1.8)
Unknown or not reported / missing	1 (1.0)	10 (6.8)	4 (2.4)	1 (1.0)	1 (2.9)	13 (4.6)
**Ethnicity**						
Hispanic	10 (9.9)	19 (12.9)	24 (14.3)	10 (9.9)	1 (2.9)	42 (15.0)
Non‐Hispanic	91 (90.1)	126 (85.7)	142 (84.5)	91 (90.1)	34 (97.1)	234 (83.6)
Unknown	0 (0.0)	2 (1.4)	2 (1.2)	0 (0.0)	0 (0.0)	4 (1.4)
**Missing teeth**, n (SD)	0.6 (1.2)	1.5 (1.5)	1.6 (1.5)	0.6 (1.2)	1.2 (1.4)	1.6 (1.6)
**PD**, mm, mean (SD)	1.7 (0.7)	2.3 (1.2)	2.9 (1.5)	1.7 (0.7)	2.2 (1.0)	2.6 (1.4)
**CAL**, mm, mean (SD)	1.1 (0.8)	2.1 (1.4)	2.6 (1.7)	1.1 (0.8)	1.7 (0.9)	2.5 (1.6)
**Plaque**, % of sites (SD)	50.8 (24.7)	66.4 (21.7)	71.9 (22.8)	50.8 (24.7)	64.9 (20.5)	69.9 (22.6)
**BOP**, % of sites (SD)	19.6 (19.1)	36.0 (20.0)	56.4 (24.6)	19.6 (19.1)	37.5 (23.4)	48.1 (24.7)

Abbreviations: BOP, bleeding on probing; CAL, clinical attachment loss; PD, probing depth.

**TABLE 2:** Baseline demographic and clinical characteristics of periodontitis progression groups.

Parameter	No progression	With 1–2 sites progressing	With ≥3 sites progressing	With 6 sites w/cumulative CAL ≥2 mm
Entire sample, 368 (row %)	186 (50.5)	97 (26.4)	48 (13.0)	37 (10.1)

	*n* (col %)	*n* (col %)	*n* (col %)	*n* (col %)
**Sex**				
Male	69 (37.1)	37 (38.1)	23 (47.9)	18 (48.6)
**Age**				
Years, mean (SD)	46.8 (13.5)	49.6 (11.7)	47.6 (14.1)	53.3 (13.6)
**Race**				
African American	38 (20.4)	26 (26.8)	12 (25.0)	13 (35.1)
White	128 (68.8)	56 (57.3)	25 (52.1)	21 (56.8)
Asian	12 (6.5)	7 (7.2)	6 (12.5)	1 (2.7)
American Indian / Alaskan Native	1 (0.5)	0 (0.0)	0 (0.0)	1 (2.7)
More than 1 race	4 (2.2)	1 (1.0)	1 (2.1)	1 (2.7)
Unknown or not reported / missing	3 (1.6)	7 (7.2)	4 (8.3)	0 (0.0)
**Ethnicity**				
Hispanic	22 (11.8)	12 (12.4)	6 (12.5)	7 (18.9)
Non‐Hispanic	163 (87.6)	85 (87.6)	40 (83.3)	29 (78.4)
Unknown	1 (0.5)	0 (0.0)	2 (4.2)	1 (2.7)
**Missing teeth**, *n* (SD)	1.2 (1.5)	1.5 (1.7)	1.4 (1.4)	1.9 (1.5)
**PD**, mm, mean (SD)	2.2 (1.2)	2.5 (1.3)	2.5 (1.4)	2.9 (1.7)
**CAL**, mm, mean (SD)	1.9 (1.3)	2.1 (1.5)	2.2 (1.7)	2.9 (1.9)
**Plaque**, % of sites (SD)	59.1 (24.6)	62.5 (23.6)	76.0 (17.1)	75.5 (22.5)
**BOP**, % of sites (SD)	35.0 (25.9)	39.8 (23.2)	48.3 (25.8)	60.8 (26.0)

Abbreviations: BOP, bleeding on probing; CAL, clinical attachment loss; PD, probing depth.

**FIGURE 1:**
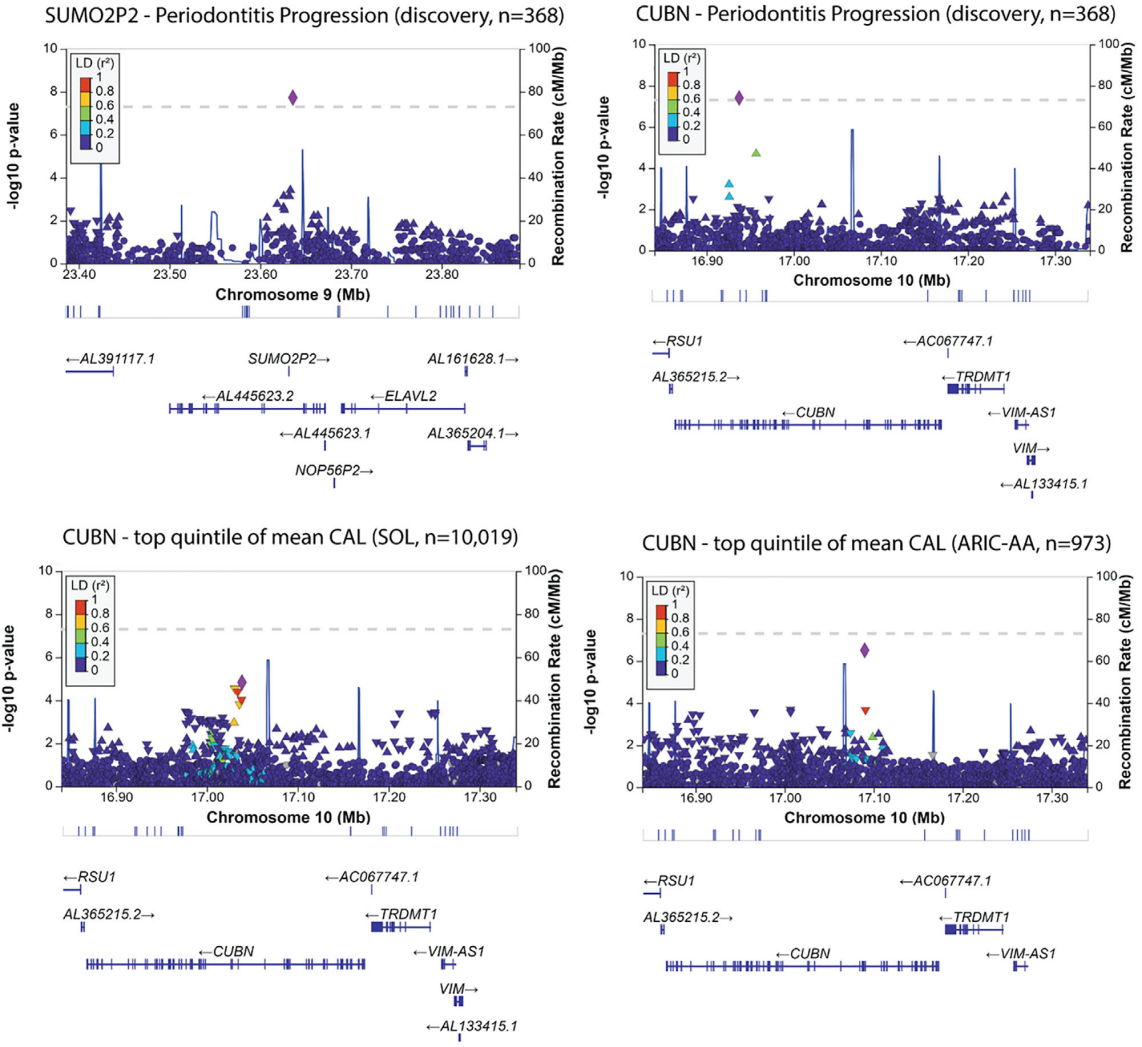
Regional association plots for the 2 loci demonstrating genome‐wide significant evidence of association with periodontitis progression and evidence of association of the CUBN locus in the independent samples of 973 African Americans (ARIC‐AA)^23^ and 10,019 Hispanic/Latinos (HCHS/SOL).^22^

In this study, genomic inflation was well‐controlled (Figure S1 in the online Journal of Periodontology), and all GWAS SNPs collectively explained about 1/3 (*h* ^2 ^= 0.34, standard error = 0.26, *p *= 0.08) of phenotypic variance in severe periodontitis and more than half (*h* ^2 ^= 0.57, standard error = 0.26, *p *= 0.01) of variance in disease progression (Table 3). Single‐marker analyses revealed 2 genome‐wide significant loci associated with disease progression, and 11 more loci with suggestive evidence of association (*p* < 10^−6^) with these 2 traits (Table 4). The 2 genome‐wide significant loci were *SUMO2P2* (small ubiquitin like modifier 2), on chromosome 9, nominated by the relatively rare rs72691774 [minor allele frequency (MAF) = 1%; *p* = 1.9x10^−8^] and *CUBN* (cubilin), on chromosome 10, nominated by the intronic rs565051161 [minor allele frequency (MAF) = 4%; *p* = 3.9x10^−8^] (Figure 1).

**TABLE 3:** Phenotypic variance explained (i.e., heritability, *h* ^2^) in periodontal disease progression and severity by all GWAS SNPs using Genome‐Wide Complex Trait Analysis (GCTA).

	Disease progression	Severe vs. mild/no disease	Stage III vs. Stage II/no disease
	*n* = 368	*n *= 416	*n* = 416
*h* ^2^ (std. error)	0.57 (0.26)	0.34 (0.26)	0.00 (0.27)
*p*‐Value	0.01	0.08	0.50

Abbreviation: GCTA, Genome‐wide Complex Trait Analysis.

Note: p‐values obtained via restricted maximum likelihood estimation (REML) implemented in GCTA.

**TABLE 4:** Loci demonstrating the strongest evidence of genome‐wide association (*p* < 10^−6^) with severe periodontitis and disease progression and information of the lead SNP in each locus.

Trait	Locus	chr	SNP rsid	EA	OA	MAF	OR/beta	se	*p*‐Value	Function	CADD; RDB
Periodontitis progression	*SUMO2P2*	9	rs72691774	C	T	0.01	1.84	0.32	1.9x10^−8^	intergenic	0.6; 7
Periodontitis progression	*CUBN*	10	rs565051161	T	C	0.04	1.15	0.21	3.9x10^−8^	intronic	1.3; 5
Periodontitis progression	*NXPH1*	7	rs114029027	A	G	0.02	1.76	0.33	1.5x10^−7^	intergenic	9.2; 7
Periodontitis progression	*CTC‐507E12.1*	5	rs185440614	C	A	0.01	1.61	0.30	1.8x10^−7^	intergenic	0.1; 5
Periodontitis progression	*AC073257.2*	2	rs13395537	C	T	0.02	1.73	0.33	1.9x10^−7^	intergenic	0.1; 5
Severe periodontitis	*ZBTB16:RP11‐64D24.2*	11	rs454802	C	T	0.45	0.46	0.15	2.2x10^−7^	ncRNA, intronic	0.2; N/A
Periodontitis progression	*IL33*	9	rs79199203	A	T	0.01	1.68	0.32	3.0x10^−7^	intronic	1.4; 6
Periodontitis progression	*MTND1P10*	22	22:36576329:C:CG	C	CG	0.03	1.40	0.27	3.1x10^−7^	downstream	2.0; N/A
Periodontitis progression	*MDN1*	6	rs201350197	A	AT	0.07	0.76	0.18	4.0x10^−7^	intronic	0.6; N/A
Periodontitis progression	*AC068483.1*	2	rs1866471	A	G	0.03	1.02	0.20	4.0x10^−7^	intergenic	1.9; N/A
Periodontitis progression	*RNA5SP472*	19	rs143325027	C	T	0.01	1.56	0.31	5.4x10^−7^	intergenic	5.2; 7
Periodontitis progression	*CTD‐2341M24.1*	14	rs17736859	G	A	0.01	1.23	0.24	7.4x10^−7^	intergenic	13.5; 7
Periodontitis progression	*DNAH17*	17	rs57713956	C	T	0.10	0.67	0.13	8.1x10^−7^	intronic	0.2; 6

*Note*: Only the SUMO2P2 and CUBN loci met genome‐wide statistical significance criteria (*p* < 5x10^−8^).

Abbreviations: CADD, Combined Annotation Dependent Depletion score (higher is predicted to be more deleterious); EA, effect allele; OA, other allele; OR, odds ratio; RDB, Regulome Database score (lower score indicates more evidence of SNP having a regulatory role); se, standard error; SNP, single nucleotide polymorphism.

These 2 polymorphisms were rare, elevating the possibility of being false positives. Indeed, we did not find any evidence of generalization for the *SUMO2P2* locus in the external cohorts. Conversely, the *CUBN* locus provided the strongest genome‐wide association signal for high CAL among the independent sample of African Americans (lead SNP: rs7082270, *p* = 3.1x10^−7^) and was also strongly associated with the same trait among Hispanic/Latinos (lead SNP: rs1276710, *p *= 1.5x10^−5^), albeit the lead SNPs differed between the 3 populations (Figure 1). The lead SNP in this study (rs565051161) was not found in the external genotype data and a nearby SNP (rs2137426) in linkage disequilibrium (LD = 0.8) was associated with tooth loss (*p* = 5x10^−3^, meeting the Bonferroni‐corrected threshold of *p* < 0.05/6 = 0.008 for this trait) among Hispanic/Latinos (Table S2 in the online *Journal of Periodontology*).

Although not genome‐wide significant, the locus with strongest evidence of association with severe periodontitis was on *ZBTB16* (zinc finger and BTB domain containing 16) on chromosome 11, marked by the intronic rs454802 (MAF = 45%, *p* = 2.2x10^−7^), overlapping with the non‐coding transcript RP11‐64D24.2 (Figure S2 in the online *Journal of Periodontology*). Of note, this locus also harbors the correlated variant rs375620 which has considerable functional implications, that is, CADD score = 20.4, indicative of being in the top 1% of variants in terms of having functional implications, and Regulome DB score 3a, demonstrating some evidence of transcription factor binding.

Gene‐centric analyses revealed no genes with genome‐wide evidence of association (*p* < 2.5x10^−6^), while 6 genes (including 1 pseudogene, the non‐coding AP000721.4, in the severe periodontitis analysis) met the suggestive evidence of association threshold (Table 5). These included *NAA40* (N‐alpha‐acetyltransferase 40, NatD catalytic subunit; *p* = 2.8x10^−6^), *COX8A* (cytochrome c oxidase subunit 8A; *p* = 2.9x10^−5^), *RRP12* (ribosomal RNA processing 12 homolog; *p* = 7.1x10^−5^) that emerged in the severe periodontitis analysis and *LTBR* (lymphotoxin beta receptor; *p* = 3.1x10^−5^), and *SPRYD4* (SPRY domain containing 4; *p* = 4.0x10^−5^) that emerged in the disease progression analysis. Secondary analyses involving the WW18 stages‐based classification did not reveal any noteworthy findings and were largely null—heritability was estimated to be practically 0, and there were no single markers of genes demonstrating genome‐wide or even suggestive evidence of association.

**TABLE 5:** Genes with the strongest evidence of gene‐centric association (*p* < 10^−4^) in severe periodontitis and disease progression GWAS.

Gene symbol	Gene name	Trait	Gene‐centric p	Description
NAA40	N‐alpha‐acetyltransferase 40, NatD catalytic subunit	Severe periodontitis	2.8x10^−6^	Enables H2A histone acetyltransferase activity; H4 histone acetyltransferase activity; and peptide‐serine‐N‐acetyltransferase activity. Involved in N‐terminal protein amino acid acetylation; histone H2A acetylation; and histone H4 acetylation. Located in centriolar satellite; cytosol; and nucleoplasm
COX8A	Cytochrome c oxidase subunit 8A	Severe periodontitis	2.9x10^−5^	The gene encodes for the terminal enzyme of the respiratory chain, coupling the transfer of electrons from cytochrome c to molecular oxygen, with the concomitant production of a proton electrochemical gradient across the inner mitochondrial membrane.
LTBR	Lymphotoxin beta receptor	Periodontitis progression	3.1x10^−5^	This gene encodes a member of the tumor necrosis factor receptor superfamily. The major ligands of this receptor include lymphotoxin alpha/beta and tumor necrosis factor ligand superfamily member 14. The encoded protein plays a role in signaling during the development of lymphoid and other organs, lipid metabolism, immune response, and programmed cell death. Activity of this receptor has also been linked to carcinogenesis.
AP000721.4	Non‐coding sequence	Severe periodontitis	6.6x10^−5^	N/A
RRP12	Ribosomal RNA processing 12 homologs	Severe periodontitis	7.1x10^−5^	Enables RNA binding activity. Predicted to be involved in rRNA processing. Located in cytosol; nucleolus; and plasma membrane.
SPRYD4	SPRY domain containing 4	Periodontitis progression	4.0x10^−5^	May play a role as a tumor suppressor gene and may be involved in regulating cell growth and apoptosis.

Abbreviation: GWAS, genome‐wide association study.

The examination of previously reported periodontitis‐associated SNPs and loci was largely negative in this study, with no markers meeting replication criteria after multiple testing correction. Four loci, *FTO* (rs8047395)^27^, *AKAP6* (rs17522122)^27^ (Table S3 in the online *Journal of Periodontology*), *PLG* (rs2465836, rs1247559),^4^ and *IL37* (rs3811047)^26^ (Table S4, Figure S3 in the online *Journal of Periodontology*) showed nominal evidence of association (*p* < 0.05) in this study.

## 4 DISCUSSION

This GWAS was conducted among a small but well‐characterized sample of adults who underwent comprehensive periodontal examinations at baseline and then were followed longitudinally for at least 6 visits during a 12‐month period to document disease progression. The rich clinical data enabled the identification of robust data‐driven disease progression groups among this population, that allowed for the conduct of a GWAS of periodontitis progression. Remarkably, we found that over half of variance in disease progression over a 12‐month period was explainable by all GWAS SNPs in this study. We also identified 2 genome‐wide significant loci (*SUMO2P2* and *CUBN*) associated with periodontitis progression, emphasizing that these associations must be treated conservatively given the rarity of these polymorphisms and the study's small sample size. Nevertheless, *CUBN* showed strong evidence of association with CAL in 2 independent community‐based samples African Americans and Hispanic/Latinos. While this association must be further replicated and mechanistically validated, we posit that it offers a promising candidate for future investigations.

The *CUBN*‐encoded cubilin facilitates the uptake of vitamin B12 which is essential for DNA and protein synthesis, energy production and fat metabolism. Recent reports implicate variations in *CUBN* with cardiovascular disease risk,^28^ as well as kidney function and proteinuria.^29^ While this is the first direct report of an association between *CUBN* and periodontitis, it is crucial to point out that lower serum vitamin B12 levels were associated with periodontal disease progression (i.e., increases in PD and CAL) and incident tooth loss in a cohort of German adults who were followed for a 6‐year period.^30^ Even less is known about *SUMO2P2*, a pseudogene that has been robustly implicated in educational attainment and related cognitive traits in a genome‐wide association study of 3 million individuals.^31^ While previously considered nonfunctional, this locus is hypothesized to regulate neuronal plasticity via the SUMOylation pathway, potentially acting as a competing endogenous RNA.

The only locus with suggestive evidence of association with the dichotomous severe periodontitis trait was *ZBTB16* (zinc finger and BTB domain containing 16), a transcriptional repressor of *NLRP7* and inflammasome activity is associated with blood neutrophil counts. Importantly, ZBTB16 has been shown to promote the osteoblastic differentiation of mesenchymal stem cells as an up‐stream regulator of *RUNX2*,^32^ and has been implicated in bone resorption (i.e., is upregulated) during orthodontic tooth movement^33^.

The results of this study must be considered while acknowledging its limitations. The obvious shortcoming of this report is its reliance on a small sample size of approximately 400 adults for a GWAS. Additionally, the lead SNPs in the 2 identified loci were relatively rare, elevating the likelihood of false positives, and the one nominating the *CUBN* locus was not found in the replication cohorts. However, proxy and adjacent (i.e., intronic) SNPs in the same loci did provide strong evidence of association for periodontal traits, adding confidence to the relevance of *CUBN* in periodontitis susceptibility. Nevertheless, the locus cannot be considered validated unless mechanistic evidence of association is added to these observational associations.

While acknowledging these limitations, we posit that the report contains useful information that adds to the knowledge base of periodontal genomics and can be used by the periodontal research community for replication of other findings and the conduct of meta‐analyses. For example, *PLG* ^4^,^34^ and *IL37* ^26^ are loci with known associations and mechanistically validated roles in periodontitis, and both showed nominal evidence of association in this study—similar to the previously reported *FTO* and *AKAP6* polymorphisms loci that were identified as shared between periodontitis and type 2 diabetes^27^. While based on a small sample size, the study benefits from the application of rigorous criteria for the selection of periodontitis cases and controls and the unusual opportunity to formally interrogate the genomic basis of periodontitis progression in an untreated study sample.

## 5 CONCLUSION

This study identified that over half of variance in periodontitis progression over a 12‐month period in an untreated cohort of adults may be explainable by genomics, i.e., individual susceptibility. Moreover, the study adds new candidate loci for interrogation as periodontitis susceptibility loci for severe periodontitis and periodontitis progression—these results should be treated conservatively and examined for independent replication and potential mechanistic implications in future studies.

## AUTHOR CONTRIBUTIONS

*Substantial contributions to the conception or design of the work*: Kimon Divaris and Flavia Teles. *Contribution to data analysis*: Camila Furquim, Ganesh Chandrasekaran, Kevin Moss, Michael J. Kallan, Andrew J. Cucchiara, Joseph Glessner, Poojan Shrestha, Kimon Divaris, and Flavia Teles. *Contribution to the data collection and/or development of the cohort studies included in the analysis*: Lynn Martin, Michele Patel, Kari E. North, Kimon Divaris, Flavia Teles, and James D. Beck. *Interpretation of data for the work*: Kimon Divaris and Flavia Teles. *Drafting the work or revising it critically for important intellectual content*: Kimon Divaris and Flavia Teles. Final approval of the version to be published: all authors reviewed and contributed to the final manuscript.

## CONFLICT OF INTEREST STATEMENT

The authors declare no conflicts of interest.

## Supporting information



Supporting Information

Supporting Information

Supporting Information

Supporting Information

Supporting Information

Supporting Information

Supporting Information

## Data Availability

The data supporting the findings of this study are not publicly available. Access to the data may be possible upon reasonable request to the corresponding author and subject to approval, but may be limited due to legal, ethical, or institutional guidelines.

## Footnotes

* PCPUNC 15, Hu‐Friedy Co., Chicago, IL

† BD Vacutainer, Becton, Dickinson and Company, Franklin Lakes, NJ

‡ Illumina Infinium Global Screening Array‐24 v3.0 BeadChip, Illumina, Inc., San Diego, CA
