# Supplementary figures and images for: Genome‐wide association study of periodontitis severity and progression
Source: J Periodontol. 2025 Dec 17;97(2):247–58. doi: 10.1002/jper.70017 (PMC13001132; doi:10.1002/jper.70017)

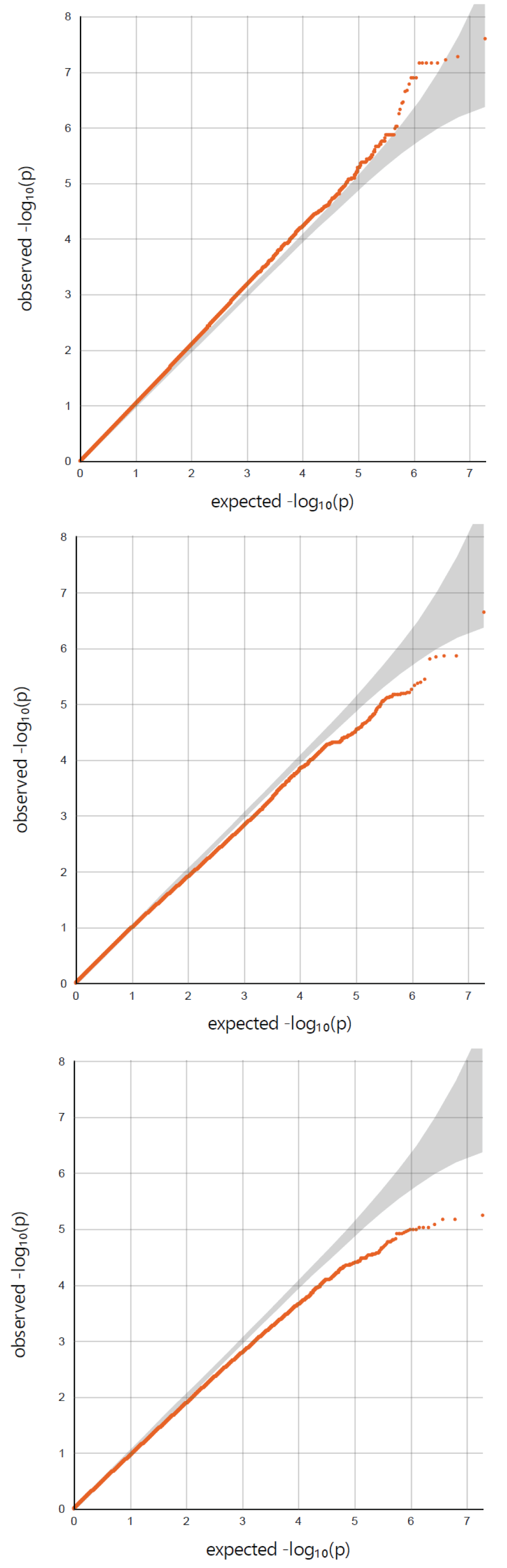

Supplement: Supplementary file 1 — Supporting Information [file JPER-97-247-s004.png]

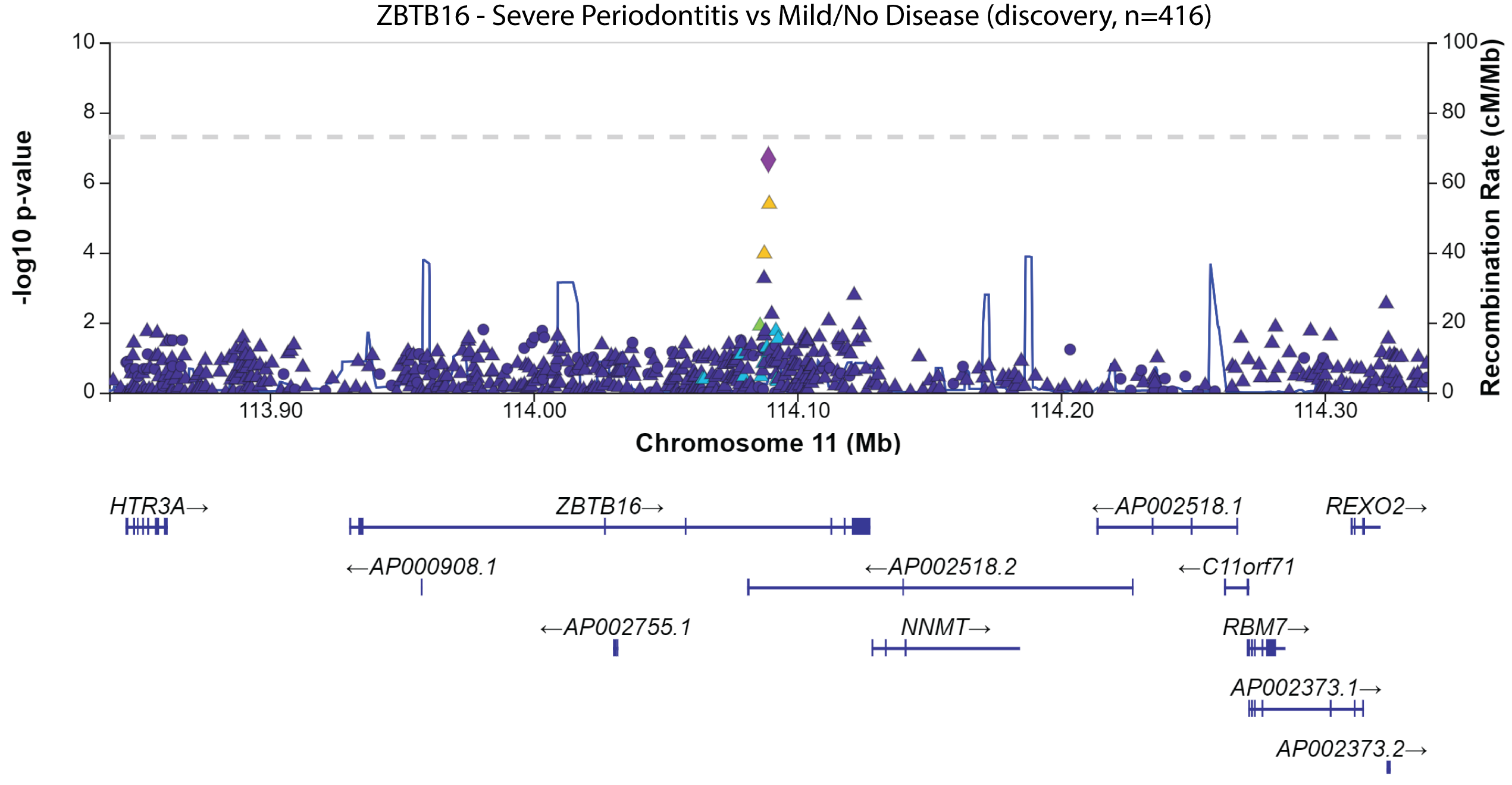

Supplement: Supplementary file 2 — Supporting Information [file JPER-97-247-s005.png]

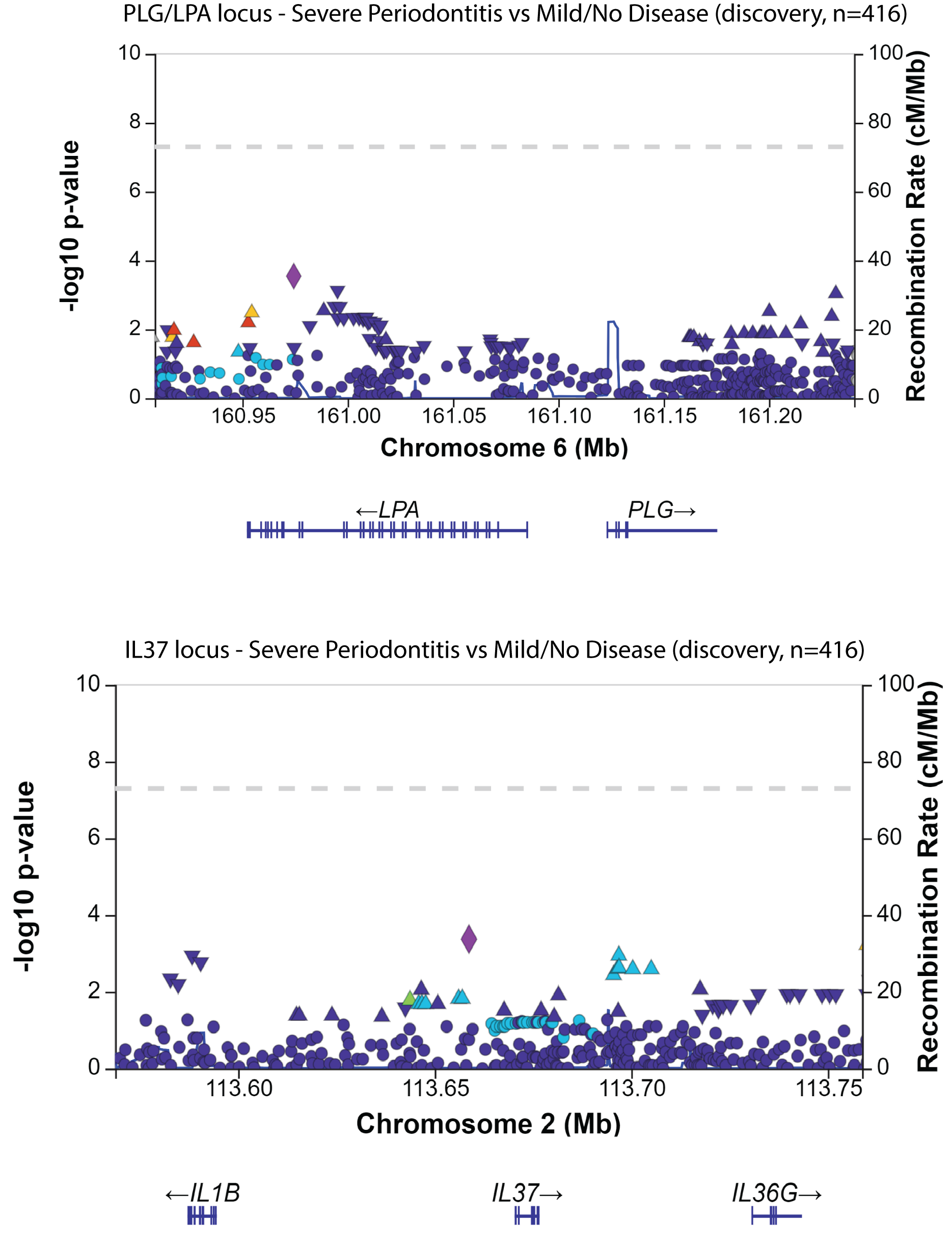

Supplement: Supplementary file 3 — Supporting Information [file JPER-97-247-s007.png]
