# Supplementary material for: Genome‐wide association study of periodontitis severity and progression
Source: J Periodontol. 2025 Dec 17;97(2):247–58. doi: 10.1002/jper.70017 (PMC13001132; doi:10.1002/jper.70017)
Supplement: Supplementary file 4 — Supporting Information [file JPER-97-247-s003.docx]

| **Supplemental Table 1**. Descriptive information of study participants at the baseline visit. | |
| --- | --- |
|  | n (%) |
| Entire sample | 416 (100.0) |
| **Sex** |  |
| male | 176 (42.3) |
|  |  |
| **Age** |  |
| years, mean (SD) | 48.4 (13.2) |
| **Race** |  |
| African American | 102 (24.6) |
| White | 262 (63.0) |
| Asian | 27 (6.5) |
| American Indian / Alaskan Native | 2 (0.5) |
| More than one race | 8 (1.9) |
| Unknown/not reported/missing | 15 (3.6) |
| **Ethnicity** |  |
| Hispanic | 53 (12.7) |
| Non-Hispanic | 359 (86.3) |
| Unknown | 4 (1.0) |
| **Periodontitis severity** |  |
| none | 101 (24.3) |
| mild | 147 (35.3) |
| severe | 168 (40.4) |
| **Periodontal Disease Stage** ^13^ |  |
| none | 101 (24.3) |
| Stage II | 35 (8.4) |
| Stage III | 280 (67.3) |
| **Periodontal Disease Progression** ^14^ |  |
| 0 sites progressing | 186 (44.7) |
| 1-2 sites progressing | 97 (23.3) |
| ≥3 sites progressing | 48 (11.5) |
| 6 sites w/cumulative CAL ≥2 mm | 37 (8.9) |
| *missing* | 48 (11.5) |

**References**

13. Papapanou PN, Sanz M, Buduneli N, et al. Periodontitis: Consensus report of workgroup 2 of the 2017 World Workshop on the Classification of Periodontal and Peri-Implant Diseases and Conditions. J Periodontol. 2018;89 Suppl 1:S173-S182. doi: 10.1002/JPER.17-0721.

14. Teles R, Benecha HK, Preisser JS, et al. Modelling changes in clinical attachment loss to classify periodontal disease progression. J Clin Periodontol. 2016;43(5):426-434. doi:10.1111/jcpe.12539
