# Supplementary material for: Genome‐wide association study of periodontitis severity and progression
Source: J Periodontol. 2025 Dec 17;97(2):247–58. doi: 10.1002/jper.70017 (PMC13001132; doi:10.1002/jper.70017)
Supplement: Supplementary file 5 — Supporting Information [file JPER-97-247-s002.docx]

| **Supplemental Table 2**. Summary of evidence of generalization of the two loci (*SUMO2P2* and *CUBN*) with genome-wide evidence of association with periodontitis progression in the external cohorts of European Americans, African Americans (ARIC study) and Hispanic/Latinos (HCHS/SOL study). | | | | | | | | | | | | | | | | |
| --- | --- | --- | --- | --- | --- | --- | --- | --- | --- | --- | --- | --- | --- | --- | --- | --- |
| Loci identified as genome-wide significantly associated with periodontitis progression in the current study | | | | | European Americans (n=4,554) ^23^ | | | | African Americans  (n=973) ^23^ | | | | Hispanic/Latinos  (n=10,019) ^22^ | | | |
|  |  |  |  |  | CAL | | tooth loss | | CAL | | tooth loss | | CAL | | tooth loss | |
| *locus* | SNP | MAF | OR | p | b | p | b | p | b | p | b | p | b | p | b | p |
| *SUMO2P2* | rs72691774 | 0.01 | 1.84 | 1.9x10^-8^ |  |  | 0.27 | 0.09 | 0.14 | 0.62 | -0.17 | 0.23 | -0.15 | 0.42 | 0.02 | 0.82 |
| *CUBN* | rs565051161 | 0.04 | 1.15 | 3.9x10^-8^ |  |  |  |  |  |  |  |  |  |  |  |  |
|  | rs2137426* | 0.004 |  |  |  |  | 0.07 | 0.73 | 0.03 | 0.75 | 0.07 | 0.16 | 0.05 | 0.51 | 0.09 | 5x10^-3^ |
| *Selected as proxy of rs565051161, in linkage disequilibrium (LD=0.80). Blank cells represent unobtainable results (i.e., nominating SNP not found in replication cohorts or not estimable result). | | | | | | | | | | | | | | | | |

**References**

22. Sanders AE, Sofer T, Wong Q, et al. Chronic periodontitis genome-wide association study in the Hispanic Community Health Study / Study of Latinos. J Dent Res. 2017;96(1):64-72. doi:10.1177/0022034516664509

23. Divaris K, Monda KL, North KE, et al. Exploring the genetic basis of chronic periodontitis: a genome-wide association study. Hum Mol Genet. 2013;22(11):2312-2324. doi:10.1093/hmg/ddt065
