# Supplementary material for: Genome‐wide association study of periodontitis severity and progression
Source: J Periodontol. 2025 Dec 17;97(2):247–58. doi: 10.1002/jper.70017 (PMC13001132; doi:10.1002/jper.70017)
Supplement: Supplementary file 6 — Supporting Information [file JPER-97-247-s006.docx]

| **Supplemental Table 3**. Association results of loci reported as genome-wide significantly associated (p<5x10^-8^) with periodontitis traits in the GWAS Catalog. | | | | | |
| --- | --- | --- | --- | --- | --- |
| GWAS Catalog Information | | Position (GRCh37) | Present Study Association Results | | |
| Risk allele | P |  | Disease progression | Severe Periodontitis | Stage III vs Stage II/Healthy ^13^ |
| rs75933965-A | 6.0x10^-13^ | 10:114749421 | 0.05 | 0.49 | 0.91 |
| rs8047395-A | 2.0x10^-12^ | 16:53798523 | 0.52 | **0.03** | 0.09 |
| rs12255678-T | 6.0x10^-11^ | 10:114729482 | 0.92 | 0.95 | 0.77 |
| rs149290349-A | 3.0x10^-10^ | 2:43451957 | 0.92 | 0.88 | 0.76 |
| rs10770140-T | 8.0x10^-10^ | 11:2193597 | 0.36 | 0.38 | 0.91 |
| rs76895963-T | 2.0x10^-9^ | 4:113006939 | 0.87 | 0.93 | 0.05 |
| rs4376068-A | 2.0x10^-9^ | 3:185497635 | 0.54 | 0.99 | 0.61 |
| rs1537415-G | 6.0x10^-9^ | 9:138529722 | 0.06 | 0.48 | 0.40 |
| rs77464186-A | 8.0x10^-9^ | 11:72460398 | 0.21 | 0.99 | 0.26 |
| rs3200401-T | 9.0x10^-9^ | 11:65271832 | 0.50 | 0.61 | 0.41 |
| rs729876-T | 1.0x10^-8^ | 16:13388778 | 0.39 | 0.42 | 0.84 |
| rs17522122-T | 1.0x10^-8^ | 14:33302882 | 0.22 | **0.01** | **0.04** |
| rs242016-A | 2.0x10^-8^ | 12:3788260 | 0.38 | 0.61 | 0.71 |
| rs2010390-A | 2.0x10^-8^ | 8:9047178 | 0.13 | 0.27 | 0.65 |
| rs2546494-A | 3.0x10^-8^ | 17:46959525 | 0.47 | 0.16 | 0.80 |
| rs6711375-A | 3.0x10^-8^ | 2:161090873 | 0.95 | 0.42 | 0.80 |
| rs1265758-A | 3.0x10^-8^ | 6:32323529 | 0.64 | 0.49 | 0.46 |
| rs11084095-A | 5.0x10^-8^ | 19:52127030 | 0.47 | 0.87 | 0.28 |
| rs8047395 is intron variant, *FTO* locus, MAF(G)=0.44  rs17522122 is a 3’ UTR variant, *AKAP6* locus, MAF (T)=0.45  boldface signifies nominal statistical significance (p<0.05) | | | | | |

**Reference**

13. Papapanou PN, Sanz M, Buduneli N, et al. Periodontitis: Consensus report of workgroup 2 of the 2017 World Workshop on the Classification of Periodontal and Peri-Implant Diseases and Conditions. J Periodontol. 2018;89 Suppl 1:S173-S182. doi: 10.1002/JPER.17-0721.
