# Supplementary material for: Genome‐wide association study of periodontitis severity and progression
Source: J Periodontol. 2025 Dec 17;97(2):247–58. doi: 10.1002/jper.70017 (PMC13001132; doi:10.1002/jper.70017)
Supplement: Supplementary file 7 — Supporting Information [file JPER-97-247-s001.docx]

| **Supplemental Table 4**. Association results of loci previously reported in the literature as associated with periodontitis and not included in the current GWAS catalog or accompanied with mechanistic validation. | | | | | |
| --- | --- | --- | --- | --- | --- |
| GWAS Catalog Information | | Position (GRCh37) | Present Study Association Results | | |
| Risk allele | locus |  | Disease progression | Severe Periodontitis | Stage III vs Stage II/Healthy ^13^ |
| rs4252200-A | *PLG* | 6:161175721 | 0.58 | 0.90 | 0.85 |
| rs1806449-T | *PLG* | 6:161280932 | 0.14 | 0.68 | 0.11 |
| rs14224-T | *PLG* | 6:161137779 | 0.53 | 0.31 | 0.90 |
| rs783145-G | *PLG* | 6:161187044 | 0.69 | 0.40 | 0.89 |
| rs2465836-G | *PLG* | 11:2193597 | 0.77 | **0.02** | 0.31 |
| rs1247559-C | *PLG* | 6:161203756 | 0.86 | **0.03** | 0.11 |
| rs2070902-T | *FCER1G* | 1:161187665 | 0.69 | 0.51 | 0.88 |
| rs10988663-T | *HMNC2* | 9:133028095 | N/A | N/A | N/A |
| rs150956098-G | *ZNF248* | 10:38173479 | N/A | N/A | N/A |
| rs75527084-C | *LINC00355* | 13:64818746 | N/A | N/A | N/A |
| rs7224672-G | *MIR4522* | 17:25548742 | 0.08 | 0.56 | 0.22 |
| rs72832278-A | *GPR179* | 17:36490427 | N/A | N/A | N/A |
| rs11084094-C | *SIGLEC5* | 19:52119546 | 0.65 | 0.20 | 0.58 |
| rs3811046 | *IL37* | 2:113671378 | 0.06 | 0.75 | 0.07 |
| rs3811047 | *IL37* | 2:113671410 | 0.05 | 0.94 | **0.03** |
| rs2708943 | *IL37* | 2:113674709 | 0.06 | 0.07 | 0.28 |
| boldface signifies nominal statistical significance (p<0.05) | | | | | |

**Reference**

13. Papapanou PN, Sanz M, Buduneli N, et al. Periodontitis: Consensus report of workgroup 2 of the 2017 World Workshop on the Classification of Periodontal and Peri-Implant Diseases and Conditions. J Periodontol. 2018;89 Suppl 1:S173-S182. doi: 10.1002/JPER.17-0721.
